# Pulmonary Recruitment Maneuver for Reducing Shoulder Pain after Laparoscopic Gynecologic Surgery: A Network Meta-Analysis of Randomized Controlled Trials

**DOI:** 10.1155/2020/7154612

**Published:** 2020-07-20

**Authors:** Chumnan Kietpeerakool, Siwanon Rattanakanokchai, Aranya Yantapant, Ratchadaporn Roekyindee, Songphol Puttasiri, Marut Yanaranop, Jatupol Srisomboon

**Affiliations:** ^1^Department of Obstetrics and Gynecology, Faculty of Medicine, Khon Kaen University, 123 Mitraparb Road, Amphur Muang, Khon Kaen 40002, Thailand; ^2^Department of Obstetrics and Gynecology, Rajavithi Hospital, College of Medicine, Rangsit University, Phayathai Road, Ratchathewi District, Bangkok 10400, Thailand; ^3^Department of Epidemiology and Biostatistics, Faculty of Public Health, Khon Kaen University, 123 Mitraparb Road, Amphur Muang, Khon Kaen 40002, Thailand; ^4^Department of Obstetrics and Gynecology, Faculty of Medicine, Chiang Mai University, 110 Inthawarorot St, Muang District, Muang Chiang Mai, Chiang Mai 50200, Thailand

## Abstract

**Background:**

Shoulder pain is a common symptom following laparoscopic surgery. This systematic review was undertaken to assess updated evidence regarding the effectiveness and complications of the pulmonary recruitment maneuver (PRM) for reducing shoulder pain after laparoscopic gynecologic surgery.

**Methods:**

A number of databases for randomized controlled trials (RCTs) investigating PRM for reducing shoulder pain were searched up to June 2019. Two authors independently selected potentially relevant RCTs, extracted data, assessed risk of bias, and compared results. Network meta-analyses were employed to simultaneously compare multiple interventions. Effect measures were presented as pooled mean difference (MD) or risk ratio (RR) with corresponding 95% confidence intervals (CI).

**Results:**

Of the 44 records that we identified as a result of the search (excluding duplicates), eleven RCTs involving 1111 participants were included. Three studies had an unclear risk of selection bias. PRM with a maximum pressure of 40 cm H_2_O was most likely to result in the lowest shoulder pain intensity at 24 hours (MD −1.91; 95% CI −2.06 to −1.76) while PRM with a maximum pressure of 40 cm H_2_O plus intraperitoneal saline (IPS) appeared to be the most efficient at 48 hours (MD −2.09; 95% CI −2.97 to −1.21). The estimated RRs for analgesia requirement, nausea/vomiting, and cardiopulmonary events were similar across the competing interventions.

**Conclusion:**

PRM with 40 cm H_2_O performed either alone or accompanied by IPS is a promising intervention for alleviating shoulder pain within 48 hours following gynecologic laparoscopy.

## 1. Introduction

Shoulder pain is a common symptom following laparoscopic gynecologic surgery. However, the exact mechanism of laparoscopy-induced shoulder pain remains unclear. Residual carbon dioxide (CO_2_) trapped between the liver and the right diaphragm is believed to be an irritation of the diaphragm, leading to referred C4 dermatomal shoulder pain [1]. Another possible pathophysiology of shoulder pain after laparoscopic surgery is that intraperitoneal carbonic acid derived from residual CO_2_ may induce irritation to the phrenic nerve. Approximately 20%–80% of patients undergoing laparoscopic gynecologic surgery experience shoulder pain [1, 2]. Not only is shoulder pain sometimes more severe than surgical wound pain, it also cannot be effectively alleviated by analgesia; thus, it may delay recovery and hospital discharge [1, 2]. Effective intervention for reducing shoulder pain following laparoscopic surgery, therefore, is mandatory.

A pulmonary recruitment maneuver (PRM) is the procedure carried out to increase positive airway pressure by performing manual pulmonary inflations. The PRM increases intrathoracic pressure and, therefore, facilitates removal of carbon dioxide from the peritoneal cavity. The PRM is simple and requires no additional equipment or medication; thus, it appears to be the most promising intervention for reducing shoulder pain after gynecologic laparoscopic surgery [3, 4].

This systematic review and network meta-analysis was undertaken to assess the updated evidence regarding the effectiveness and harms of PRM for reducing shoulder pain after laparoscopic gynecologic surgery in the following issues: (1) magnitudes of effect measures of PRM compared to usual practice; (2) comparison between PRM alone and a combination of PRM with other intervention(s); and (3) appropriate maximum inspiratory pressure to be used for PRM.

## 2. Methods

This meta-analysis was performed and reported in compliance with the PRISMA statement [5]. The details of the protocol for this systematic review were registered with PROSPERO (CRD42019140427).

### 2.1. Criteria for Considering Studies for This Review

This review included randomized controlled trials (RCTs) irrespective of the language of publication, year of publication, or sample size. The population was women who underwent any kind of gynecologic laparoscopy. Intervention of interest was PRM at any maximum inflation pressure performed alone or in combination with another intervention. Comparisons included abdominal compression aimed at expelling as much residual CO_2_ as possible, PRM alone with different inflation pressures from that applied in the intervention group, or any combination of PRM with another intervention. Crossover trials and cluster-randomized trials were invalid in this context.

### 2.2. Types of Outcome Measures

The primary outcome was the intensity of shoulder pain evaluated at 24 hours following operation as this is a typical time of most intense pain. Secondary outcomes included shoulder pain intensity at 48 hours, incidence of shoulder pain at 24 and 48 hours, postoperative nausea/vomiting, cardiopulmonary complications, and requirement of postoperative analgesia.

### 2.3. Search Methods for Identification of Studies

To identify potential eligible studies, a systematic literature search was conducted in the major electronic databases including MEDLINE, PubMed, Scopus, ISI Web of Science, Cochrane Central Register of Controlled Trials (CENTRAL), CINAHL, and LILACS from their inception to June 2019 (Supplement Table S1). The titles of all relevant articles were identified on Google Scholar, and then a further search related to these studies was performed focusing on the first 50 records identified [6]. The World Health Organization International Clinical Trials Registry Platform (http://www.who.int/ictrp/en/) and ClinicalTrials.gov, to identify ongoing trials, were searched. OpenGrey (http://www.opengrey.eu/) was searched for grey literature (Supplement Table S1). The citation lists of included studies, key textbooks, and previous reviews were also assessed.

### 2.4. Study Selection and Data Extraction

Titles and abstracts of studies retrieved by electronic searching were screened independently by two authors (CK and SR). Those studies that clearly did not satisfy the inclusion criteria were excluded. The full texts of potentially eligible studies were retrieved and independently assessed by two authors (CK and SR). Any disagreement between them over the eligibility of particular studies was resolved through discussion. Two authors (CK and SR) independently extracted data from the included studies using a data extraction form that was designed and pilot‐tested.

### 2.5. Risk of Bias Assessment

The Cochrane Risk of Bias Tool for Randomized Controlled Trials [7] to assess the quality of included studies was applied. CK and SR independently evaluated risk of bias of each included study in the following aspects: (1) random sequence generation (selection bias); (2) allocation concealment (selection bias); (3) blinding of participants and personnel (performance bias); (4) blinding of outcome assessment (detection bias); (5) completeness of outcome data (attrition bias); and (6) selective outcome reporting (reporting bias). The risk of bias in each item was categorized as low, high, and unclear [7].

### 2.6. Statistical Analysis

A random-effects network meta-analysis was employed to simultaneously compare multiple interventions for each outcome. Abdominal compression or abdominal pressure was used as the common comparator in the network model. Network meta-analysis followed the steps recommended by Shim et al. [8].

The comparison of intervention for each outcome was graphically summarized as network geometry [9]. Nodes represented available intervention assessed, while links between the nodes indicated a direct comparison between pairs of interventions [9, 10]. Direct and indirect evidence from any pair of interventions were combined to generate mixed treatment effect sizes. The global inconsistency test was applied to assess the inconsistency between direct and indirect estimates by a fitting design-by-intervention inconsistency model. Significant heterogeneity was considered if *p* value was less than 0.05 [11].

Effect measures were presented as pooled mean differences (MD) with corresponding 95% confidence intervals (CIs) for the continuous outcome (i.e., shoulder pain score) and risk ratio (RR) for binary outcomes (i.e., incidence of shoulder pain). If continuous outcomes of any included studies were expressed as median and range, the authors were contacted to obtain the outcome means and standard deviations (SD). If this was not possible, the data were converted to mean and SD using the method proposed by Wan et al. [12]. The surface under the cumulative ranking area (SUCRA) was applied to rank the hierarchy of effective interventions for the intensity of shoulder pain [7].

Prespecified subgroup analysis was carried out to determine the impact of the procedure complexity on the intensity and incidence of shoulder pain. A study with major procedure complexity was defined as the one where uterine surgery including hysterectomy and myomectomy accounted for more than 50% of the operations performed. Alternatively, it was defined as a study with minor procedure complexity.

Prespecified sensitivity analysis was done for shoulder pain intensity and incidence of shoulder pain 24 hours after operation to assess the robustness of the finding by restricting analyses to trials with low risk of selection bias.

All analyses were performed using STATA version 15.1 (StataCorp, College Station, TX, USA).

## 3. Results

### 3.1. Characteristics of Included Studies

**Figure 1:**
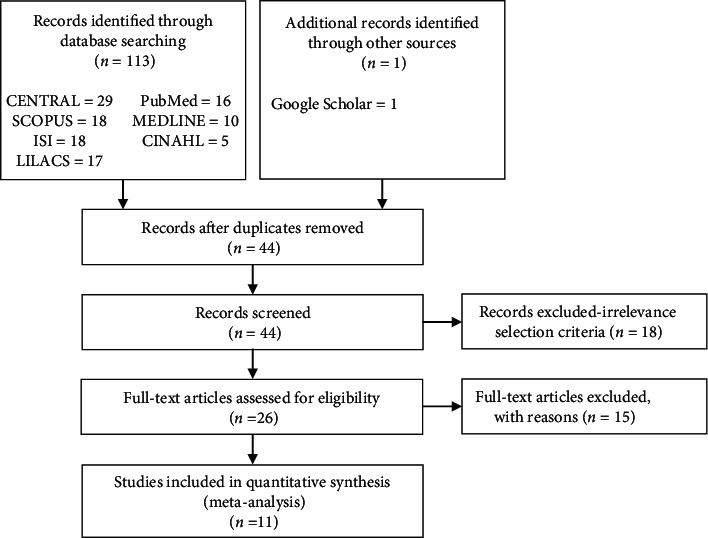
PRISMA flow diagram.

A broad search in June 2019 yielded 113 references from electronic databases. One additional reference was identified through Google Scholar. After deduplication, we screened titles and abstracts of 44 references and excluded 17 that obviously did not meet the review inclusion criteria. Of the 27 references that potentially satisfied inclusion criteria, 16 references included three ongoing studies that were excluded after reviewing the details of the study. Finally, 11 studies for the analyses were included (Supplement Tables S2–S5).  Figure 1 displays the PRISMA flowchart for study selection.

Five included studies compared PRM with maximum inspiratory pressure (varying from 30 to 60 cm H_2_O) to abdominal compression [13–17]. A comparison of four included studies was a combination of PRM and intraperitoneal saline (IPS) versus abdominal compression [1, 18–20]. One study compared PRM 40 cmH20 with IPS to PRM 60 cmH20 with IPS [21]. One study compared three interventions including PRM, IPS, and abdominal compression. Only the results of comparison between PRM and abdominal compression were included [22]. Ten studies were conducted in participants with benign gynecologic conditions [1, 13–15, 17–22]. Only one study assessed the effect of PRM following laparoscopic surgery for gynecologic cancer [16].

### 3.2. Risk of Bias in Included Studies

**Figure 2:**
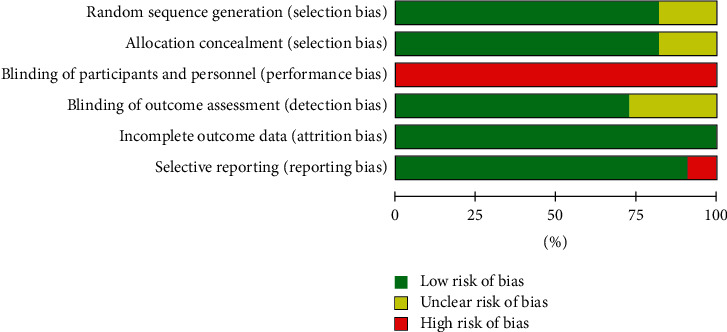
Summary risk of bias of included studies.

Figure 2 shows the risk of bias representing the overall risk of bias of included studies. All included studies had a high risk of performance bias as they were unable to blind the personnel to the intervention assigned. Three included studies were determined as unclear risk of bias in terms of random sequence generation and allocation concealment. None of them had evidence of a definite high risk of selection (Supplement Figure S1).

### 3.3. Effects of Interventions

**Figure 3:**
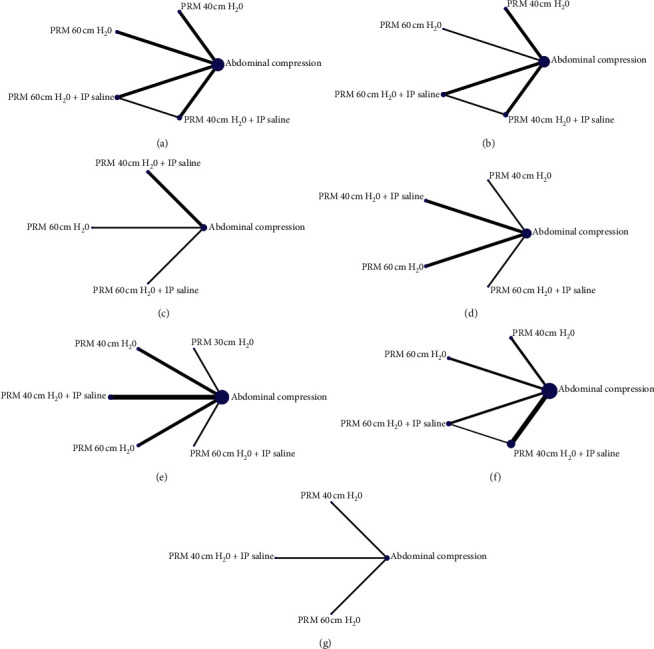
Network geometry of outcomes (Abd: abdominal compression, PRM 40: pulmonary recruitment maneuver 40 cm H_2_O, PRM 60: pulmonary recruitment maneuver 60 cm H_2_O, IP saline: intraperitoneal saline): (a) intensity of shoulder pain at 24 hours, (b) intensity of shoulder pain at 48 hours, (c) incidence of shoulder pain at 24 hours, (d) incidence of shoulder pain at 48 hours, (e) postoperative nausea/vomiting, (f) cardiopulmonary complications, and (g) requirement of postoperative analgesia.

Network diagrams of all the eligible comparisons are presented in Figure 3.

### 3.4. Intensity of Shoulder Pain at 24 Hours

Seven studies involving 726 participants determined shoulder pain intensity at 24 hours after the operation [1, 13, 14, 16, 18, 21, 22]. When compared with abdominal compression, either PRM alone or PRM combined with IPS significantly reduced shoulder pain intensity with MD of VAS varying from 1.5 to 1.9. There was no significant benefit of IPS for reducing shoulder pain intensity at 24 hours when combined with PRM. Participants undergoing PRM at a maximum inspiratory pressure of 60 cm H_2_O experienced a slightly higher shoulder pain than those who underwent PRM performed with a maximum inspiratory pressure of 40 cm H_2_O (MD 0.42; 95% CI 0.21 to 0.63) (Table 1). The SUCRA rankings indicated that PRM with a maximum inspiratory pressure of 40 cm H_2_O was most likely to result in the lowest shoulder pain intensity at 24 hours while abdominal compression seemed appreciably less attractive than the other alternatives (Table 2 and Supplement Figure S2).

### 3.5. Intensity of Shoulder Pain at 48 Hours

Six studies involving 626 participants assessed this outcome [1, 14, 16, 18, 21, 22]. At 48 hours following the operation, participants receiving a combination of PRM and IPS reported less intensity of shoulder pain than those who underwent abdominal compression. There was no significant difference in shoulder pain score between the participants who received abdominal compression and those who received PRM regardless of maximum inspiratory pressure applied (Table 1). The effect of PRM in reducing shoulder pain intensity at 48 hours did not differ across the maximum inspiratory pressure of 40 and 60 cmH20. The SUCRA rankings showed that a combination of PRM and IPS appeared to be the most promising option at this time point (Table 2 and Supplement Figure S2).

### 3.6. Incidence of Shoulder Pain at 24 and 48 Hours

Among the available comparisons evaluating the risk of developing shoulder pain at 24 hours after the operation, a combination of PRM and IPS can reduce the risk of shoulder pain with estimated RRs ranging from 0.69 (95% CI 0.51–0.95) to 0.66 (95% CI 0.46–0.93) depending on maximum inspiratory pressure applied. For the 48-hour time point, only PRM with a pressure of 60 cm H_2_O was noted to be a marginal significant intervention associated with lower risk of shoulder pain (RR 0.81; 95% CI 0.66–0.99) (Table 1).

### 3.7. Postoperative Nausea/Vomiting and Cardiopulmonary Complications

**Figure 4:**
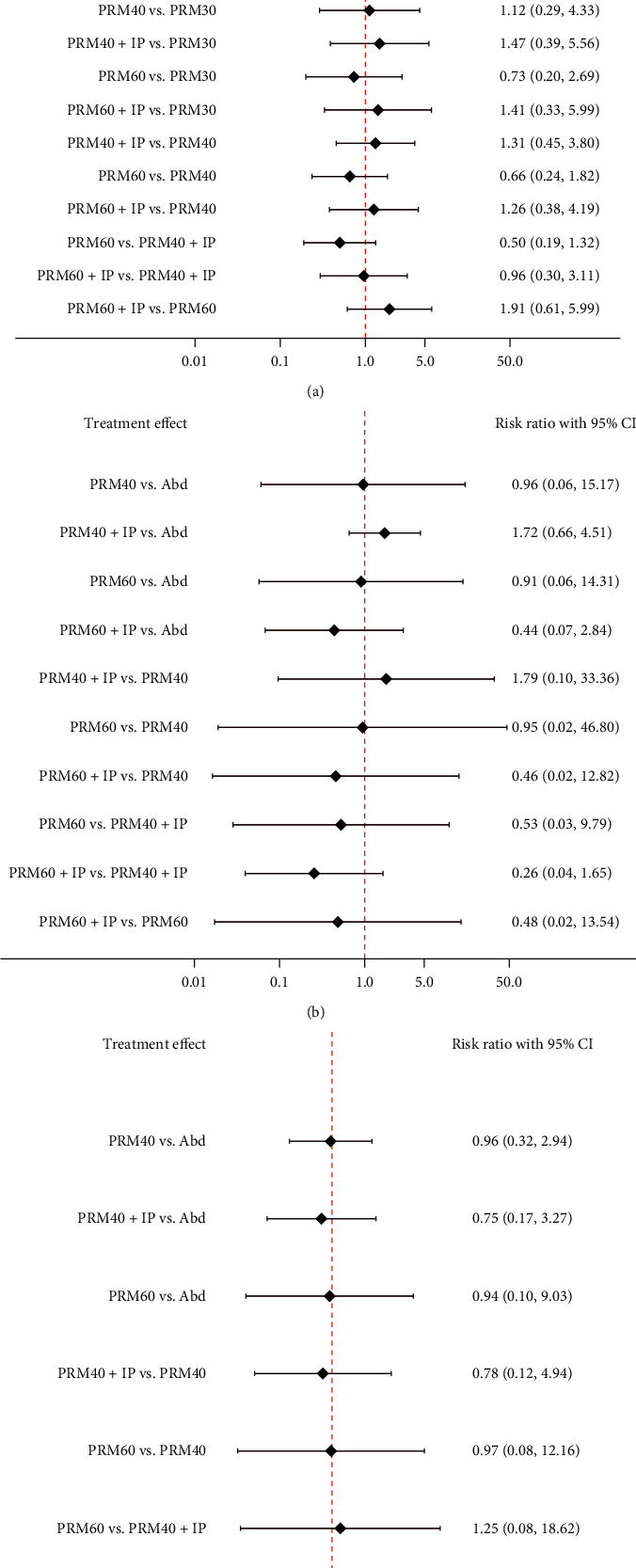
Network meta-analyses of adverse outcomes and the requirement of postoperative analgesia—risk ratio with 95% confidence interval. Abd: abdominal compression, PRM 40: pulmonary recruitment maneuver 40 cm H_2_O, PRM 60: pulmonary recruitment maneuver 60 cm H_2_O, IP saline: intraperitoneal saline, SUCRA: surface under the cumulative ranking curve. (a) Postoperative nausea/vomiting, (b) cardiopulmonary complications, and (c) requirement of postoperative analgesia.

The estimated RRs of all available interventions for secondary outcomes are presented in Figure 4. All competing interventions showed similar risks of postoperative nausea/vomiting and cardiopulmonary complications.

### 3.8. Requirement of Postoperative Analgesia

Estimated RRs of the postoperative analgesic requirement were similar across the different comparisons (Figure 4).

### 3.9. Subgroup and Sensitivity Analyses

Significant reduction of pain intensity after the application of PRM alone or PRM combined with IPS was shown across the studies with different complexities of laparoscopic procedures when compared to abdominal compression (Supplement Table S6). The reduction of shoulder pain intensity after an application of PRM was still robust in the trials with a low risk of selection bias (Supplement Table S7).

Three outcomes including shoulder pain intensity at 24 hours and 48 hours and cardiopulmonary complications were technically feasible to be tested for global inconsistency which indicated no evidence of inconsistency of treatment effects (Supplement Table S8).

## 4. Discussion

Updated evidence from this review is based on eleven RCTs involving 1111 participants. This systematic review shows that PRM alone or in combination with IPS effectively reduced shoulder pain intensity compared to abdominal compression. There was no evidence supporting the use of high maximum inspiratory pressure for PRM, as the effectiveness in reducing shoulder pain intensity among participants who underwent PRM performed with a maximum inspiratory pressure of 60 cm H_2_O appeared to be similar to those with a PRM with a maximum pressure of 40 cm H_2_O. Subgroup analysis revealed the benefits of either PRM alone or in combination with IPS in reducing shoulder pain across the studies with different levels of surgical complexity. The robustness of the review findings was reaffirmed by sensitivity analyses. Based on this updated evidence, PRM at a maximum pressure of 40 cm H_2_O performed either alone or accompanied by IPS was the most promising intervention for alleviating shoulder pain within 48 hours following gynecologic laparoscopy.

In 2003, Taş et al. [3] reported results of a systematic review which evaluated the interventions to alleviate shoulder pain after laparoscopic surgery for the benign gynecologic condition. This review included three RCTs for assessing the effectiveness of PRM alone compared to abdominal compression or IPS. This review proposed that PRM using a pressure of 40 cm H_2_O is a simple and cost-effective method to reduce shoulder pain after laparoscopy for benign gynecological conditions [3].

The second systematic review to investigate the effectiveness of PRM in reducing shoulder pain occurring after laparoscopic surgery was published by Pergialiotis et al. [4]. This review included five RTCs in which four RCTs were conducted among women undergoing laparoscopic gynecologic surgery. Comparisons in this review included PRM alone versus abdominal compression (four studies) and PRM combined with IPS versus abdominal compression (one study). The authors noted that PRM performed either alone or in combination with IPS significantly reduced the intensity of shoulder pain at 12, 24, and 48 hours [4].

Recently, Kaloo et al. [23] updated their Cochrane review which was conducted to assess the interventions to reduce shoulder pain following gynecological laparoscopic procedures. Control in all comparisons in this review was abdominal compression. The authors of this review assessed several interventions using numerous separate pairwise meta-analyses. This review included five and three RCTs for evaluating the incidence of shoulder pain at 72 hours and shoulder pain intensity at 24 hours among women undergoing PRM compared to those who received abdominal compression. This review noted that participants undergoing PRM experienced lower shoulder pain intensity than those who received abdominal compression although there was no significant difference in the incidence of shoulder pain among the comparison groups [23].

An updated finding of the present review, which is based on eleven RCTs involving 1111 participants, reaffirmed that PRM performed either alone or accompanied by IPS significantly reduced shoulder pain intensity compared to abdominal compression. Nevertheless, there are important differences between the previous systematic reviews and the present review. Firstly, this review was not restricted to studies that used abdominal compression as a treatment for the controls. Secondly, in an attempt to yield unbiased pooled estimates, this review focused on gynecologic laparoscopic surgery in order to minimize the impact of effect modifiers secondary to differences in characteristics of surgical procedures and disease severity. Thirdly, a network meta-analysis, which allowed the simultaneous analysis of both direct and indirect comparisons among various interventions across multiple RCTs, was applied. By means of network meta-analysis, the present review was able to provide additional insights into the results of existing systematic reviews in that there was no evidence supporting the use of 60 cm H_2_O high maximum inspiratory pressure for PRM, as the effectiveness in reducing shoulder pain intensity was similar to that performed by a low-pressure PRM. In addition, SUCRA rankings indicated that PRM with a maximum inspiratory pressure of 40 cm H_2_O was most likely to result in the lowest shoulder pain intensity at the 24-hour assessment while a combination of PRM and IPS seemed to be the most effective intervention at 48 hours. Finally, the present review also addressed the safety issues of PRM which have never been reported in the previous reviews.

Less well studied is whether low inspiratory pressure PRM is as effective as higher pressure for reducing postlaparoscopic shoulder pain. Ryu et al. [21] compared PRM that applied a maximum pressure of 40 cm H_2_O with IPS to PRM at a pressure of 60 cm H_2_O with IPS and noted no significant differences in the intensity of shoulder pain at 24 and 48 hours between the two comparison groups. Interestingly, Lee et al. [17] compared PRM at a maximum pressure of 30 cm H_2_O to abdominal compression and observed a significant reduction of shoulder pain score at 24 and 48 hours among participants assigned to treatment by low-pressure PRM. Numerical data regarding the intensity and incidence of shoulder pain measured in a study of Lee et al. [17], however, could not be meta-analyzed in the present review. Based on the present network meta-analysis, in which only comparison of PRM with pressures of 40 cm H_2_O to 60 cm H_2_O was available to assess, the low-pressure PRM was as effective as a high-pressure PRM (Table 1). More studies are needed to confirm the beneficial effects of PRM when performed with a maximum pressure of lower than 40 cm H_2_O.

Interestingly, SUCRA rankings indicated that PRM with a maximum inspiratory pressure of 40 cm H_2_O was most likely to result in the lowest shoulder pain intensity at 24-hour assessment while a combination of PRM and IPS seemed to be the most efficient at 48 hours. This difference might be secondary to the fact that PRM is a direct mechanical technique to evacuate the residual CO_2_ in the abdominal cavity. In contrast, intraperitoneal saline instillation is a physiologic intervention. Instilled saline acts as a dissolved buffer to alleviate peritoneal irritation from carbonic acid. The effect of PRM alone, therefore, might not persist for as long as that of a combination of PRM and IPS [1, 22].

The major strength of this review is that these findings were derived from network meta-analyses which allowed quantitative comparisons of interventions that have not been directly compared in primary studies. This information is pivotal for clinical decision making particularly in the absence of head-to-head evidence [24]. In addition, gathering both direct and indirect evidence collectively strengthens the inference concerning the relative effect measure of competing interventions that have been carried out in a limited number of primary studies. The probability of one treatment being the best for reducing shoulder pain intensity at each specific time point through SUCRA rankings, was also estimated. This review has some limitations. As it was not feasible to blind the personnel involved in the study, i.e., surgeon and anesthesiologist, to intervention received, all included studies were deemed as having a high risk of performance bias. A limited number of included studies precluded the ability to create comparison-adjusted funnel plots to assess the small-study effects.

In conclusion, PRM is a promising intervention to reduce shoulder pain occurring after laparoscopic gynecologic surgery. This updated evidence suggests that PRM with 40 cm H_2_O performed either alone or in combination with IPS should be the intervention of first choice for alleviating shoulder pain within 48 hours following gynecologic laparoscopy. More studies are needed to explore the effects of PRM performed with a maximum pressure of lower than 40 cm H_2_O.

## Figures and Tables

**Table 1:** Network-estimated effect measures of intervention on shoulder pain.

Pain scores of shoulder pain at 24 hours—mean difference of VAS (95% confidence intervals)				
Abdominal compression				
−1.91 (−2.06, −1.76)	PRM 40 cm H_2_O			
−1.75 (−2.48, −1.02)	0.16 (−0.58, 0.90)	PRM 40 cm H_2_O plus IPS		
−1.49 (−1.64, −1.34)	0.42 (0.21, 0.63)	0.26 (−0.48, 1.00)	PRM 60 cm H_2_O	
−1.53 (−2.28, −0.78)	0.38 (−0.38, 1.15)	0.22 (−0.69, 1.13)	−0.04 (−0.80, 0.72)	PRM 60 cm H_2_O plus IPS

Scores of shoulder pain at 48 hours—mean difference of VAS (95% confidence intervals)				
Abdominal compression				
−0.58 (−1.20, 0.05)	PRM 40 cm H_2_O			
−2.09 (−2.97, −1.21)	−1.52 (−2.60, −0.44)	PRM 40 cm H_2_O plus IPS		
−0.26 (−1.55, 1.03)	0.32 (−1.12, 1.75)	1.83 (0.28, 3.39)	PRM 60 cm H_2_O	
−1.46 (−2.36, −0.55)	−0.88 (−1.98, 0.22)	0.64 (−0.42, 1.70)	−1.20 (−2.77, 0.37)	PRM 60 cm H_2_O plus IPS

Incidence of shoulder pain at 24 hours—risk ratio (95% confidence intervals)				
Abdominal compression				
—	PRM 40 cm H_2_O			
0.69 (0.51, 0.95)	—	PRM 40 cm H_2_O plus IPS		
0.91 (0.70, 1.18)	—	1.31 (0.88, 1.96)	PRM 60 cm H_2_O	
0.66 (0.46, 0.93)	—	0.95 (0.59, 1.51)	0.72 (0.47, 1.11)	PRM 60 cm H_2_O plus IPS

Incidence of shoulder pain at 48 hours—risk ratio (95% confidence intervals)				
Abdominal compression				
0.77 (0.58, 1.03)	PRM 40 cm H_2_O			
0.73 (0.48, 1.11)	0.95 (0.57, 1.58)	PRM 40 cm H_2_O plus IPS		
0.81 (0.66, 0.99)	1.05 (0.74, 1.50)	1.11 (0.69, 1.77)	PRM 60 cm H_2_O	
0.60 (0.36, 1.00)	0.78 (0.44, 1.40)	0.82 (0.42, 1.58)	0.74 (0.43, 1.28)	PRM 60 cm H_2_O plus IPS

VAS: visual analogue scale (0–10); PRM 40: pulmonary recruitment maneuver 40 cm H_2_O; PRM 60: pulmonary recruitment maneuver 60 cm H_2_O; IPS: intraperitoneal saline.

**Table 2:** Ranking of the interventions using SUCRA scores.

SUCRA score (%)	Abd	PRM 40	PRM 40 plus IPS	PRM 60	PRM 60 plus IPS
Score of shoulder pain					
24 hours	0.0	87.7	69.2	42.6	50.5
48 hours	9.8	42.2	96.5	26.7	74.8

Abd: abdominal compression; PRM 40: pulmonary recruitment maneuver 40 cm H_2_O; PRM 60: pulmonary recruitment maneuver 60 cm H_2_O; IPS: intraperitoneal saline; SUCRA: surface under the cumulative ranking curve.

## Supplementary Materials

Supplementary Materials Supplement Table S1: search strategy and results. Supplement Table S2: characteristics of excluded studies. Supplement Table S3: characteristics of included studies. Supplement Table S4: number of studies and participants of each outcome. Supplement Table S5: reported outcomes of included studies. Supplement Figure S1: risk of bias of each included study. Supplement Figure S2: SUCRA ranking of the interventions. Supplement Table S6: subgroup analysis according to complexity of procedures. Supplement Table S7: sensitivity by excluded unclear and high risk of bias study on random sequence generation and allocation concealment bias. Supplement Table S8: evaluation of the global inconsistency in network meta-analysis.
