# Supplementary material for: Pulmonary Recruitment Maneuver for Reducing Shoulder Pain after Laparoscopic Gynecologic Surgery: A Network Meta-Analysis of Randomized Controlled Trials
Source: Minim Invasive Surg. 2020 Jul 20;2020:7154612. doi: 10.1155/2020/7154612 (PMC7387971; doi:10.1155/2020/7154612)
Supplement: Supplementary Materials — Supplement Table S1: search strategy and results. Supplement Table S2: characteristics of excluded studies. Supplement Table S3: characteristics of included studies. Supplement Table S4: number of studies and participants of each outcome. Supplement Table S5: reported outcomes of included studies. Supplement Figure S1: risk of bias of each included study. Supplement Figure S2: SUCRA ranking of the interventions. Supplement Table S6: subgroup analysis according to complexity of procedures. Supplement Table S7: sensitivity by excluded unclear and high risk of bias study on random sequence generation and allocation concealment bias. Supplement Table S8: evaluation of the global inconsistency in network meta-analysis. [file 7154612.f1.docx]

**Supplement Information**

**Pulmonary recruitment maneuver for reducing shoulder pain after laparoscopic gynecologic surgery: A network meta-analysis of randomized controlled trials**

**Supplement Table S1: search strategy and results**

**PUBMED search June 22, 2019**

| **Search** | **Query** | **Item found** |
| --- | --- | --- |
| #1 | Laparoscopy [MH] | 92,445 |
| #2 | (Laparoscop* OR Celioscop* OR Peritoneoscop*) OR ((Laparoscopic) AND (Surgical OR Surger* OR Assisted OR hysterectomy)) | 134,646 |
| #3 | Hysteroscopy [MH] | 4,584 |
| #4 | Hysteroscop* OR Uterine Endoscop* OR Uteroscop* OR (Hysteroscopic AND Surger*) | 7,586 |
| #5 | LH OR LAVH OR LASH | 55,704 |
| #6 | endometrial ablation techniques [MH] | 364 |
| #7 | Endometrial AND Ablation* | 1,555 |
| #8 | #1 OR #2 OR #3 OR #4 OR #5 OR #6 OR #7 | 196,061 |
| #9 | Gynecologic Surgical Procedures [MH] | 79,929 |
| #10 | (“GYN” OR gyne* or gynae*) | 545,825 |
| #11 | Gynecologic* | 71,177 |
| #12 | Ovariectomy [MH] | 24,655 |
| #13 | Ovariectom* OR Oophorectom* OR Female Castration* | 43,130 |
| #14 | Salpingostomy [MH] | 502 |
| #15 | Salpingostom* | 776 |
| #16 | Uterine Myomectomy [MH] | 797 |
| #17 | Uterine Myomectom* OR Myomectom* OR Fibroidectom* | 3,546 |
| #18 | Hysterectomy [MH] | 29,604 |
| #19 | Hysterectom* | 46,425 |
| #20 | menorrhagia OR metrorrhagia OR (uter* AND bleed*) | 20,973 |
| #21 | (benign OR endometr* OR uter* OR cervi* OR ovar* OR vagin* OR fallopian* OR vulva* OR gynae* OR gyne*) AND (disease* OR cancer* OR neoplas* OR neoplas* OR carcinom* OR malignan* OR tumor* OR tumour*) | 708,256 |
| #22 | #9 OR #10 OR #11 OR #12 OR #13 OR #14 OR #15 OR #16 OR #17 OR #18 OR #19 OR #20 OR #21 | 1,056,080 |
| #23 | Recruitment AND (manoeuvre* OR maneuver*) | 1,122 |
| #24 | #8 AND #22 AND #23 | 16 |

**Ovid Medline: search June 22, 2019**

| **Search** | **Query** | **Item found** |
| --- | --- | --- |
| #1 | exp Hysteroscopy/ | 4,581 |
| #2 | (Laparoscop$ or Celioscop$ or Peritoneoscop$).mp. or (Laparoscopic adj3 (Surgical or Surger$ or Assisted or hysterectomy)).tw. [mp=title, abstract, original title, name of substance word, subject heading word, floating sub-heading word, keyword heading word, organism supplementary concept word, protocol supplementary concept word, rare disease supplementary concept word, unique identifier, synonyms] | 116,433 |
| #3 | exp Hysteroscopy/ | 4,581 |
| #4 | (Hysteroscop$ or Uterine Endoscop$ or Uteroscop$ or (Hysteroscopic adj3 Surger$)).tw. | 5,710 |
| #5 | (LH or LAVH or LASH).tw. | 49,562 |
| #6 | exp Endometrial Ablation Techniques/ | 364 |
| #7 | (Endometrial adj3 Ablation$).tw. | 1,137 |
| #8 | 1 or 2 or 3 or 4 or 5 or 6 or 7 | 170,972 |
| #9 | exp Gynecologic Surgical Procedures/ | 79,878 |
| #10 | (GYN or gyne$ or gynae$).tw. | 86,405 |
| #11 | Gynecologic$.tw. | 38,530 |
| #12 | exp Ovariectomy/ | 24,636 |
| #13 | (Ovariectom$ or Oophorectom$ or Female Castration$).tw. | 33,567 |
| #14 | exp Salpingostomy/ | 502 |
| #15 | Salpingostom$.tw. | 437 |
| #16 | exp Uterine Myomectomy/ | 794 |
| #17 | (Uterine Myomectom$ or Myomectom$ or Fibroidectom$).tw. | 2,790 |
| #18 | exp Hysterectomy/ | 29,581 |
| #19 | Hysterectom$.tw. | 30,511 |
| #20 | (menorrhagia or metrorrhagia or (uter$ adj3 bleed$)).tw. | 7,541 |
| #21 | (benign or endometr$ or uter$ or cervi$ or ovar$ or vagin$ or fallopian$ or vulva$ or gynae$ or gyne$).mp. and (disease$ or cancer$ or neoplas$ or neoplas$ or carcinom$ or malignan$ or tumor$ or tumour$).tw. [mp=title, abstract, original title, name of substance word, subject heading word, floating sub-heading word, keyword heading word, organism supplementary concept word, protocol supplementary concept word, rare disease supplementary concept word, unique identifier, synonyms] | 436,435 |
| #22 | 9 or 10 or 11 or 12 or 13 or 14 or 15 or 16 or 17 or 18 or 19 or 20 or 21 | 554,733 |
| #23 | (Recruitment adj3 (manoeuvre$ or maneuver$)).tw. | 779 |
| #24 | 8 and 22 and 23 | 10 |

**CENTRAL: search June 22, 2019**

| **Search** | **Query** | **Item found** |
| --- | --- | --- |
| #1 | MeSH descriptor: [Hand-Assisted Laparoscopy] explode all trees | 12 |
| #2 | (Laparoscop* OR Celioscop* OR Peritoneoscop*) OR ((Laparoscopic) AND (Surgical OR Surger* OR Assisted OR hysterectomy)) | 18,552 |
| #3 | MeSH descriptor: [Hysteroscopy] explode all trees | 381 |
| #4 | Hysteroscop* OR Uterine Endoscop* OR Uteroscop* OR (Hysteroscopic AND Surger*) | 1,632 |
| #5 | LH OR LAVH OR LASH | 10,338 |
| #6 | MeSH descriptor: [Endometrial Ablation Techniques] explode all trees | 35 |
| #7 | Endometrial AND Ablation* | 396 |
| #8 | #1 OR #2 OR #3 OR #4 OR #5 OR #6 OR #7 | 30,059 |
| #9 | MeSH descriptor: [Gynecologic Surgical Procedures] explode all trees | 4,128 |
| #10 | (“GYN” OR gyne* or gynae*) | 55,457 |
| #11 | Gynecologic* | 11,941 |
| #12 | MeSH descriptor: [Gynecologic Surgical Procedures] explode all trees | 279 |
| #13 | Ovariectom* OR Oophorectom* OR Female Castration* | 2,112 |
| #14 | MeSH descriptor: [Salpingostomy] explode all trees | 40 |
| #15 | Salpingostom* | 81 |
| #16 | MeSH descriptor: [Uterine Myomectomy] explode all trees | 49 |
| #17 | Uterine Myomectom* OR Myomectom* OR Fibroidectom* | 756 |
| #18 | MeSH descriptor: [Hysterectomy] explode all trees | 1,726 |
| #19 | Hysterectom* | 6,834 |
| #20 | menorrhagia OR metrorrhagia OR (uter* AND bleed*) | 4,283 |
| #21 | (benign OR endometr* OR uter* OR cervi* OR ovar* OR vagin* OR fallopian* OR vulva* OR gynae* OR gyne*) AND (disease* OR cancer* OR neoplas* OR neoplas* OR carcinom* OR malignan* OR tumor* OR tumour*) | 46,621 |
| #22 | #9 OR #10 OR #11 OR #12 OR #13 OR #14 OR #15 OR #16 OR #17 OR #18 OR #19 OR #20 OR #21 | 80,787 |
| #23 | Recruitment AND (manoeuvre* OR maneuver*) | 599 |
| #24 | #8 AND #22 AND #23 | 33 |
| #25 | #8 AND #22 AND #23 in Trials | 29 |

**SCOPUS: search June 22, 2019**

| **Search** | **Query** | **Item found** |
| --- | --- | --- |
| #1 | TITLE-ABS-KEY((Laparoscop* OR Celioscop* OR Peritoneoscop*) OR ((Laparoscopic) AND (Surgical OR Surger* OR Assisted OR hysterectomy))) | 176,531 |
| #2 | TITLE-ABS-KEY(Hysteroscop* OR Uterine Endoscop* OR Uteroscop* OR (Hysteroscopic AND Surger*)) | 6,006 |
| #3 | TITLE-ABS-KEY(LH OR LAVH OR LASH) | 76,336 |
| #4 | TITLE-ABS-KEY(Endometrial AND Ablation*) | 2,235 |
| #5 | #1 OR #2 OR #3 OR #4 | 256,799 |
| #6 | TITLE-ABS-KEY((“GYN” OR gyne* or gynae*)) | 206,719 |
| #7 | TITLE-ABS-KEY(Gynecologic*) | 101,238 |
| #8 | TITLE-ABS-KEY(Ovariectom* OR Oophorectom* OR Female Castration*) | 17,105 |
| #9 | TITLE-ABS-KEY(Salpingostom*) | 1,173 |
| #10 | TITLE-ABS-KEY(Uterine Myomectom* OR Myomectom* OR Fibroidectom*) | 4,262 |
| #11 | TITLE-ABS-KEY(Hysterectom*) | 69,210 |
| #12 | TITLE-ABS-KEY(menorrhagia OR metrorrhagia OR (uter* AND bleed*)) | 42,222 |
| #13 | TITLE-ABS-KEY((benign OR endometr* OR uter* OR cervi* OR ovar* OR vagin* OR fallopian* OR vulva* OR gynae* OR gyne*) AND (disease* OR cancer* OR neoplas* OR neoplas* OR carcinom* OR malignan* OR tumor* OR tumour*)) | 894,092 |
| #14 | #6 OR #7 OR #8 OR #9 OR #10 OR #11 OR #12 OR #13 | 1,034,261 |
| #15 | TITLE-ABS-KEY(Recruitment AND (manoeuvre* OR maneuver*)) | 1,379 |
| #16 | #5 AND #14 AND #15 | 18 |

**ISI: search June 22, 2019**

| **Search** | **Query** | **Item found** |
| --- | --- | --- |
| #1 | TS=((Laparoscop* OR Celioscop* OR Peritoneoscop*) OR ((Laparoscopic) AND (Surgical OR Surger* OR Assisted OR hysterectomy))) | 126,427 |
| #2 | TS=(Hysteroscop* OR Uterine Endoscop* OR Uteroscop* OR (Hysteroscopic AND Surger*)) | 6,572 |
| #3 | TS=(LH OR LAVH OR LASH) | 31,501 |
| #4 | TS=(Endometrial AND Ablation*) | 1,371 |
| #5 | #1 OR #2 OR #3 OR #4 | 163,070 |
| #6 | TS=((“GYN” OR gyne* or gynae*)) | 285,484 |
| #7 | TS=(Gynecologic*) | 39,924 |
| #8 | TS=(Ovariectom* OR Oophorectom* OR Female Castration*) | 34,304 |
| #9 | TS=(Salpingostom*) | 375 |
| #10 | TS=(Uterine Myomectom* OR Myomectom* OR Fibroidectom*) | 3,485 |
| #11 | TS=(Hysterectom*) | 30,721 |
| #12 | TS=(menorrhagia OR metrorrhagia OR (uter* AND bleed*)) | 11,909 |
| #13 | TS=((benign OR endometr* OR uter* OR cervi* OR ovar* OR vagin* OR fallopian* OR vulva* OR gynae* OR gyne*) AND (disease* OR cancer* OR neoplas* OR neoplas* OR carcinom* OR malignan* OR tumor* OR tumour*)) | 731,436 |
| #14 | #6 OR #7 OR #8 OR #9 OR #10 OR #11 OR #12 OR #13 | 795,069 |
| #15 | TS=(Recruitment AND (manoeuvre* OR maneuver*)) | 1,406 |
| #16 | #5 AND #14 AND #15 | 18 |

**CINAHL: search June 22, 2019**

| **Search** | **Query** | **Item found** |
| --- | --- | --- |
| S1 | ((Laparoscop* OR Celioscop* OR Peritoneoscop*) OR ((Laparoscopic) AND (Surgical OR Surger* OR Assisted OR hysterectomy))) | 30,039 |
| S2 | (Hysteroscop* OR Uterine Endoscop* OR Uteroscop* OR (Hysteroscopic AND Surger*)) | 2,228 |
| S3 | (LH OR LAVH OR LASH) | 1,860 |
| S4 | (Endometrial AND Ablation*) | 544 |
| S5 | S1 OR S2 OR S3 OR S4 | 33,897 |
| S6 | ((“GYN” OR gyne* or gynae*)) | 28,496 |
| S7 | (Gynecologic*) | 12,654 |
| S8 | (Ovariectom* OR Oophorectom* OR Female Castration*) | 4,173 |
| S9 | (Salpingostom*) | 48 |
| S10 | (Uterine Myomectom* OR Myomectom* OR Fibroidectom*) | 1,091 |
| S11 | (Hysterectom*) | 9,748 |
| S12 | (menorrhagia OR metrorrhagia OR (uter* AND bleed*)) | 3,954 |
| S13 | ((benign OR endometr* OR uter* OR cervi* OR ovar* OR vagin* OR fallopian* OR vulva* OR gynae* OR gyne*) AND (disease* OR cancer* OR neoplas* OR neoplas* OR carcinom* OR malignan* OR tumor* OR tumour*)) | 91,342 |
| S14 | S6 OR S7 OR S8 OR S9 OR S10 OR S11 OR S12 OR S13 | 117,046 |
| S15 | (Recruitment AND (manoeuvre* OR maneuver*)) | 451 |
| S16 | S5 AND S14 AND S15 | 5 |

**LILACS: 17 records (http://bvsalud.org/en/) search June 22, 2019**

| (tw:(((Laparoscop* OR Celioscop* OR Peritoneoscop*) OR ((Laparoscopic) AND (Surgical OR Surger* OR Assisted OR hysterectomy))) OR (Hysteroscop* OR Uterine Endoscop* OR Uteroscop* OR (Hysteroscopic AND Surger*)) OR (LH OR LAVH OR LASH) OR (Endometrial AND Ablation*))) AND (tw:(((“GYN” OR gyne* or gynae*)) OR (Gynecologic*) OR (Ovariectom* OR Oophorectom* OR Female Castration*) OR (Salpingostom*) OR (Uterine Myomectom* OR Myomectom* OR Fibroidectom*) OR (Hysterectom*) OR (menorrhagia OR metrorrhagia OR (uter* AND bleed*)) OR ((benign OR endometr* OR uter* OR cervi* OR ovar* OR vagin* OR fallopian* OR vulva* OR gynae* OR gyne*) AND (disease* OR cancer* OR neoplas* OR neoplas* OR carcinom* OR malignan* OR tumor* OR tumour*)))) AND (tw:((Recruitment AND (manoeuvre* OR maneuver*)))) |
| --- |

**Open Grey: 0 records (http://www.opengrey.eu/) search June 22, 2019**

| (((Laparoscop$ OR Celioscop$ OR Peritoneoscop$) OR ((Laparoscopic) AND (Surgical OR Surger$ OR Assisted OR hysterectomy))) OR (Hysteroscop$ OR Uterine Endoscop$ OR Uteroscop$ OR (Hysteroscopic AND Surger$)) OR (LH OR LAVH OR LASH) OR (Endometrial AND Ablation$)) AND (((“GYN” OR gyne$ or gynae$)) OR (Gynecologic$) OR (Ovariectom$ OR Oophorectom$ OR Female Castration$) OR (Salpingostom$) OR (Uterine Myomectom$ OR Myomectom$ OR Fibroidectom$) OR (Hysterectom$) OR (menorrhagia OR metrorrhagia OR (uter$ AND bleed$)) OR ((benign OR endometr$ OR uter$ OR cervi$ OR ovar$ OR vagin$ OR fallopian$ OR vulva$ OR gynae$ OR gyne$) AND (disease$ OR cancer$ OR neoplas$ OR neoplas$ OR carcinom$ OR malignan$ OR tumor$ OR tumour$))) AND (Recruitment AND (manoeuvre$ OR maneuver$)) |
| --- |

**Supplement Table S2:** Characteristics of excluded studies

| **Study 1:** A trial to reduce referred pain in the shoulder following a laparoscopic gynecological surgery (NCT02467985) | |
| --- | --- |
| Population | 175 women, age of 18 Years to 65 Years. Inclusion criteria: physical ASA score 1-2, elective laparoscopic surgery for benign cause. Exclusion criteria: pregnant women, chronic pain syndrome or preoperative pain syndrome, narcotics consumption, chronic preoperative analgesia, alcohol or drug dependence, inability to provide telephone follow up at 48 hours, and laparoscopy not possible at the umbilical level |
| Intervention | Flow of insufflation will be set to 2-3L / min. After the intervention, the CO2 insufflation is discontinued, accessories trocars are removed under direct vision and the incisions the sites of these trocars will be closed. Patients will be placed in the Trendelenburg position 30 degrees, head tilted down. The umbilical trocar is opened. Suction is inserted into the trocar, taking care to stay inside the jacket of the trocar. Active suction gas will during lung recruitment. This manoeuvre will be performed by the anaesthesiologists who apply five subsequent forced breaths, up to 40 cm H2O pressure, taking care to maintain the insufflation last 5 sec. Once completed, the suction will be removed. The laparoscope is inserted into the trocar to verify the absence of trauma to underlying structures. |
| Control | No intervention |
| Outcomes | (Time frame: 48 hours following surgery) shoulder pain, impact of referred pain in the shoulder, intensity of abdominal pain, incidence of postoperative nausea, quality of life, incidence of abdominal pain, incidence of postoperative vomiting |
| Reason for exclusion | Ongoing study with no results provided |
| **Study 2:** Combination of a simple clinical maneuver and intraperitoneal bupivacaine for the reduction of postoperative shoulder pain in gynecologic laparoscopy: a randomized, controlled trial. | |
| Population | 287 women undergoing gynecologic laparoscopy |
| Intervention | (1) Intraperitoneal instillation of bupivacaine; (2) pulmonary recruitment maneuver; (3) combination of intraperitoneal instillation of bupivacaine and pulmonary recruitment maneuver |
| Control | Placebo |
| Outcomes | Incidence and intensity of shoulder pain and complication |
| Reason for exclusion | Information is only available as an abstract. No detailed information regarding the characteristics of participants, study methodology, and numerical data regarding the outcome measured was insufficient for meta-analyses. |
| **Study 3:** Effective Maneuver for Post-laparoscopic Shoulder Pain (NCT02811081) | |
| Population | 144 participants, aged 19-65 years, with benign gynaecologic disease, with ASA status I-II. Exclusion criteria: chronic shoulder pain, chronic epigastric pain or chronic pain syndrome, past history of pneumothorax or any pulmonary surgical history, any shoulder surgery histories, patients who required to conversion to open surgery from laparoscopic surgery, patients who required to receive incidental upper abdominal surgeries due to adhesion and injury at upper abdominal cavity, patients with inability to understand or express 10 point visual analogue scale, and pregnant women |
| Intervention | (1) Normal Saline Instillation: isotonic normal saline (20 ml/kg of body weight) was infused in the sub-diaphragmatic region at the end of surgery.  (2) Normal Saline Instillation + Pulmonary Recruitment: pulmonary recruitment maneuver was performed after instillation of isotonic normal saline (20 ml/kg of body weight) in the sub-diaphragmatic region at the end of surgery. |
| Control | Residual carbon dioxide gas was evacuated by the routine method using passive exsufflation through the port site at the end of surgery. |
| Outcomes | Shoulder pain evaluated by 10 point visual analogue scale  and Wound pain evaluated by 10 point visual analogue scale (Time Frame: after two day of procedure) |
| Reason for exclusion | This reference is a registered protocol of “Ryu KH, Lee SH, Cho EA, Kim JA, Lim GE, Song T. Comparison of impacts of intraperitoneal saline instillation with and without pulmonary recruitment maneuver on post-laparoscopic shoulder pain prevention: a randomized controlled trial. Surg Endosc 2019;33(3):870-878” which was included in this review. |
| **Study 4:** Comparison of shoulder pain reducing techniques after laparoscopy and laparoscopy assisted robotic surgeries | |
| Population | Ninety women undergoing benign and malignant disease of gynecologic conditions |
| Intervention | (1) Pulmonary recruitment maneuver; (2) Intraperitoneal warm saline instillation; and (3) Intraperitoneal drain |
| Control | Not stated |
| Outcomes | Postoperative shoulder pain at 6 and 24 hours after surgery |
| Reason for exclusion | Information is only available as an abstract. No detailed information regarding the characteristics of participants, study methodology, and numerical data regarding the outcome measured was insufficient for further analyses. |
| **Study 5:** Multi-modal analgesic technique for pain control in patients undergoing diagnostic gynecological laparoscopy: randomized controlled clinical trial | |
| Population | Fifty female patients, scheduled for diagnostic gynecologic laparoscopy were included in the study.  Exclusion criteria: younger than 18 or above 45 years old, patients with ASA ≥ III, and who were allergic or hypersensitive to amide-type local anaesthetics were excluded. Also, patients with pre-existing chronic pain disorders, or with history of alcohol or drug abuse, including opioids or tranquillisers for > 1 week preoperatively, were excluded. If the laparoscopy included any interventional procedure or was converted to an open procedure, the patient was also excluded from the study. |
| Intervention | (1) Pulmonary recruitment manoeuvre and intraperitoneal lidocaine; (2) only intraperitoneal lidocaine; and (3) only pulmonary recruitment manoeuvre |
| Control | Passive exsufflation through the port site |
| Outcomes | Postoperative pain at 1,3, and 6 hours following operation; nausea; and vomiting |
| Reason for exclusion | Time frame of outcome measures in this study did not comply with the time frame that our review aimed to evaluate (12, 24, and 48 hours after operation) |
| **Study 6:** Pulmonary recruitment maneuver affects on laparoscopic complications. | |
| Population | Not applicable |
| Intervention | Not applicable |
| Control | Not applicable |
| Outcomes | Not applicable |
| Reason for exclusion | This is a narrative review |
| **Study 7:** Pulmonary recruitment manoeuvre for reducing postoperative shoulder pain incidence after laparoscopic gynecologic surgery: a prospective randomized controlled trial (TCTR20130712001) | |
| Population | 160 women scheduled for elective laparoscopic gynecologic surgery |
| Intervention | Pulmonary recruitment manoeuvre |
| Control | Abdominal compression |
| Outcomes | Incidence and intensity of shoulder pain and analgesics (paracetamol and pethidine) requirement |
| Reason for exclusion | Information is only available as an abstract. No detailed information regarding the characteristics of participants, study methodology, and numerical data regarding the outcome measured was insufficient for analyses. |
| **Study 8:** Pulmonary recruitment maneuver for reducing postoperative shoulder pain incidence after laparoscopic gynecologic surgery (TCTR20130712001) | |
| Reason for exclusion | This is a registered protocol of study 7 which was excluded from this review due to insufficiency information. |
| **Study 9:** Pulmonary recruitment maneuver to reduce pain after laparoscopy: a meta-analysis of randomized controlled trials (NCT03026530) | |
| Population | Participants undergoing laparoscopic Roux-en-Y gastric bypass or laparoscopic sleeve gastrectomy |
| Intervention | Pulmonary recruitment maneuver |
| Control | Gentle pressing of the abdominal walls with the trocar ports open |
| Outcomes | Intensity of postoperative shoulder pain at 12, 24, and 48 hours |
| Reason for exclusion | Wrong population of interest |
| **Study 10:** Simple clinical maneuver for reducing shoulder pain following gynecologic laparoscopic surgery at Maharat Nakhonratchasima Hospital: A randomized controlled trial. | |
| Population | 104 women scheduled for elective gynecologic laparoscopic surgery |
| Intervention | Pulmonary recruitment maneuver |
| Control | Abdominal compression |
| Outcomes | Shoulder pain at 24 and 48 hours |
| Reason for exclusion | No information regarding the maximal inspiratory pressure applied for pulmonary recruitment maneuver |
| **Study 11:** The effectiveness of intraperitoneal normal saline infusion and the pulmonary recruitment maneuver to reduce postlaparoscopic shoulder pain and upper abdominal pain in elective gynecologic surgeries (IRCT 201402022576n8) | |
| Population | Female patients scheduled for elective gynecologic laparoscopic surgery with ASA I-II. Women with malignant disease were ineligible. |
| Intervention | (1) Pulmonary recruitment maneuver; (2) Intraperitoneal normal saline instillation; (3) Combination of pulmonary recruitment maneuver and intraperitoneal saline instillation |
| Control | No intervention |
| Outcomes | Postoperative pain evaluated at 2,4,6,12,24 hours after operation; nausea/vomiting and abdominal distension (evaluated at 24 hours after operation) |
| Reason for exclusion | Ongoing study, no results available |
| **Study 12:** The effect of local anesthesia and simple maneuver on shoulder pain after gynecologic laparoscopy (NCT01039441) | |
| Population | - 291 women with benign adnexa diseases which are scheduled for laparoscopic surgery. Exclusion criteria include the procedure required conversion to a laparotomy, An operative time > 3 hours, Interpretation of pain was impossible due to serious adverse effects |
| Intervention | (1) Intraperitoneal instillation of bupivacaine; (2) Pulmonary recruitment manoeuvre |
| Control | Instillation of normal saline 50ml under the diaphragm |
| Outcomes | The degree of shoulder pain after gynaecologic laparoscopy [ Time Frame: During the first 24hours after surgery; 1,6, 12, 24hr |
| Reason for exclusion | Ongoing study, no results available |
| **Study 13:** POstLAparoscopic Reduction of pain By combining intraperitoneal normal salinE And the pulmonary Recruitment maneuver (POLAR BEAR trial). RCT to estimate reduction in pain after laparoscopic surgery when using a combination therapy of intraperitoneal normal saline and the pulmonary recruitment maneuver (NTR4812) | |
| Population | Women between 18 and 65 years of age, with an ASA I-II who are scheduled to undergo an elective laparoscopic procedure with benign gynecologic indication |
| Intervention | Upper abdomen is filled with normal saline infusion with the patient in the Trendelenburg position. Then the anesthesiologist performs a standardized pulmonary recruitment maneuver |
| Control | Carbon dioxide is removed from the abdominal cavity at the end of surgery, with gentle abdominal pressure and passive exsufflation through the port sites, with open sleeve valves |
| Outcomes | The primary outcomes are the incidence and intensity of post-laparoscopic pain in the shoulder, upper abdomen and at the operation sites, at 8, 24 and 48 h after surgery. Secondary outcomes are postoperative use of analgesics, nausea, vomiting and pulmonary complications. |
| Reason for exclusion | This is a published protocol of study which was included in this review (van Dijk J, Dedden SJ, Geomini P, van Kuijk S, van Hanegem N, Meijer P, Bongers MY.  Randomised controlled trial to estimate reduction in pain after laparoscopic surgery when using a combination therapy of intraperitoneal normal saline and the pulmonary recruitment manoeuvre. BJOG;125(11):1469-1476) |
| **Study 14:** Postlaparoscopic reduction of pain by combining intraperitoneal normal saline and the pulmonary recruitment maneuver (DOI 10.1007/s10397-015-0918-0) | |
| Population | Women between 18 and 65 years, ASA classification I-II, planned for an elective laparoscopic procedure with benign gynaecologic indication |
| Intervention | Combination of intraperitoneal normal saline and the pulmonary recruitment manoeuvre |
| Control | Abdominal compressure |
| Outcomes | Incidence and intensity of postlaparoscopic pain in the shoulder, upper abdomen and at the operation sites, at 8, 24 and 48 hours after surgery, postoperative use of pain medication, nausea, vomiting and pulmonary problems |
| Reason for exclusion | This reference is an abstract presented at the 24th Annual Congress of the European Society for Gynaecological Endoscopy (ESGE), 7th -10th October 2015, Syma – Budapest – Hungary (ES24-0124; Free Communication 10; Laparoscopic Surgery). The full article has been published and included in this review (van Dijk J, Dedden SJ, Geomini P, van Kuijk S, van Hanegem N, Meijer P, Bongers MY.  Randomised controlled trial to estimate reduction in pain after laparoscopic surgery when using a combination therapy of intraperitoneal normal saline and the pulmonary recruitment manoeuvre. BJOG;125(11):1469-1476). |
| - **Study 15:** Randomized clinical trial of the influence of pulmonary recruitment maneuver on reducing shoulder pain after laparoscopy (DOI: 10.1097/AAP.0b013e3181f3582c) | |
| Population | 146 patients for minor gynaecological laparoscopy |
| Intervention | Pulmonary recruitment manoeuvre |
| Control | Traditional passive deflation of abdominal cavity |
| Outcomes | Shoulder pain intensity assessed at 4, 12, 24 and 48 h after the surgery and usage of analgesics |
| Reason for exclusion | This reference is an abstract presented at the XXIX Annual European Society of Regional Anaesthesia (ESRA) Congress 2010. The full article has been published and included in this review (Sharami SH, Sharami MB, Abdollahzadeh M, Keyvan A. Randomised clinical trial of the influence of pulmonary recruitment manoeuvre on reducing shoulder pain after laparoscopy. J Obstet Gynaecol 2010;30(5):505-10). |
| **Study 16:** Post laparoscopic pain evaluation maneuvers (CTRI/2017/07/008963) | |
| Population | Women of age 18–65 years with ASA I-II who underwent laparoscopic surgeries for  benign gynecologic conditions and infertility evaluation |
| Intervention | Intraperitoneal saline instillation with pulmonary recruitment maneuver |
| Control | Abdominal pressure |
| Outcomes | Shoulder pain, upper abdomen pain assessed at 3, 6, 12, 24 and 48 hours after operation, overall incidence of pain, adverse events i.e. nausea, vomiting, abdominal distension |
| Reason for exclusion | This reference is a registered protocol of study which was included in this review Kumari A, Rajaram S, Gupta B, Kundan M. Assessment of a combination of clinical maneuvers in evaluation of post-laparoscopic pain: a randomized clinical trial. J Obstet Gynecol India 2019; available at https://doi.org/10.1007/s13224-019-01224-4). |

**Supplement Table S3** Characteristics of included studies

| Author, year  (setting) | Treatment | n | Age | BMI | Duration of surgery | Surgical complexity |
| --- | --- | --- | --- | --- | --- | --- |
| Güngördük, 2018  (Turkey) | Abdominal compression | 52 | 48.6 (7.5) ^1^ | 26.5 (2.4) ^1^ | 118.2 (50.4) ^1^ | Major |
|  | PRM 40 cmH_2_O | 54 | 49.3 (8.1) ^1^ | 26.9 (2.1) ^1^ | 114.1 (53.1) ^1^ |  |
| Kumari, 2019  (India) | Abdominal compression | 32 | - | - | - | Minor |
|  | PRM 40 cmH_2_O + IPS | 32 | - | - | - |  |
| Lee, 2019  (Korea) | Abdominal compression | 42 | 41.0 (10.3) ^1^ | 23.7 (3.8) ^1^ | 94.9 (38.5) ^1^ | Major |
|  | PRM 30 cmH_2_O | 42 | 41.4 (9.5) ^1^ | 22.6 (3.3) ^1^ | 94.9 (33.5) ^1^ |  |
| Liu, 2014  (China) | Abdominal compression | 30 | 32.3 (5.0) ^1^ | - | 65.7 (40.4) ^1^ | Minor |
|  | PRM 40 cmH_2_O | 30 | 30.2 (3.7) ^1^ | - | 54.6 (33.7) ^1^ |  |
| Phelps, 2008  (USA) | Abdominal compression | 46 | 35.0 (1.2) ^2^ | 26.6 (0.8) ^2^ | 44.5 (2.9) ^2^ | Minor |
|  | PRM 60 cmH_2_O | 54 | 33.8 (0.9) ^2^ | 25.6 (0.8) ^2^ | 41.8 (2.9) ^2^ |  |
| Ryu K, 2017  (Korea) | Abdominal compression | 30 | 41.8 (11.3) ^1^ | 23.0 (3.7) ^1^ | 66.0 (39.0, 86.5) ^3^ | Major |
|  | PRM 40 cmH_2_O + IPS | 30 | 38.7 (9.3) ^1^ | 23.5 (4.2) ^1^ | 68.0 (47.5, 109.0) ^3^ |  |
|  | PRM 60 cmH_2_O + IPS | 29 | 39.7 (10.1) ^1^ | 22.9 (4.1) ^1^ | 56.0 (40.0, 82.0) ^3^ |  |
| Ryu KH, 2019  (Korea) | Abdominal compression | 48 | 40.0 (11.0) ^1^ | 22.9 (3.4) ^1^ | 59.0 (49.0, 78.0) ^3^ | Major |
|  | PRM 40 cmH_2_O + IPS | 48 | 40.0 (10.0) ^1^ | 23.5 (4.9) ^1^ | 59.0 (43.0, 73.0) ^3^ |  |
| Sharami, 2010  (Iran) | Abdominal compression | 64 | 27.4 (6.0) ^1^ | 26.0 (4.9) ^1^ | 27.4 (12.6) ^1^ | Minor |
|  | PRM 40 cmH_2_O | 67 | 29.0 (6.1) ^1^ | 26.9 (4.0) ^1^ | 33.1 (18.3) ^1^ |  |
| Tsai HW, 2011  (Taiwan) | Abdominal compression | 51 | 41.0 (8.1) ^1^ | - | 132.3 (43.2) ^1^ | Major |
|  | PRM 60 cmH_2_O | 53 | 42.1 (8.1) ^1^ | - | 145.9 (42.2) ^1^ |  |
| Tsai HW, 2013  (Taiwan) | Abdominal compression | 50 | 38.9 (8.5) ^1^ | 22.6 (4.0) ^1^ | 148.2 (53.5) ^1^ | Major |
|  | PRM 60 cmH_2_O + IPS | 50 | 39.7 (9.0) ^1^ | 22.7 (3.9) ^1^ | 145.5 (42.5) ^1^ |  |
| van Dijk J, 2018  (Netherlands) | Abdominal compression | 88 | 42.1 (9.2) ^1^ | 25.4 (4.0) ^1^ | 66.5 (41.0, 106.0) ^3^ | Minor |
|  | PRM 40 cmH_2_O + IPS | 89 | 43.2 (9.5) ^1^ | 26.2 (4.9) ^1^ | 89.0 (54.0, 120.0) ^3^ |  |

^1^ Mean (SD), ^2^ Mean (SE), ^3^ Median (IQR)

Abd, abdominal compression; PRM 40, pulmonary recruitment maneuver 40 cmH_2_O; PRM 60, pulmonary recruitment maneuver 60 cmH_2_O; IPS, intraperitoneal saline

**Supplement Table S4:** Number of studies and participants of each outcome

| Outcome | Number of studies | Number of participants |
| --- | --- | --- |
| Pain scores of shoulder pain |  |  |
| - 24 hours | 7 | 726 |
| - 48 hours | 6 | 626 |
| Incidence of shoulder pain |  |  |
| - 24 hours | 4 | 445 |
| - 48 hours | 6 | 605 |
| Postoperative nausea/vomiting | 9 | 891 |
| Cardiopulmonary complication | 9 | 967 |
| Postoperative analgesia requirement | 3 | 306 |

**Supplement Table S5** Reported outcomes of included studies

| Study, year | Abdominal compression | | |  | PRM30 | | |  | PRM40 | | |  | PRM40 plus IPS | | |  | PRM60 | | |  | PRM60 plus IPS | | |
| --- | --- | --- | --- | --- | --- | --- | --- | --- | --- | --- | --- | --- | --- | --- | --- | --- | --- | --- | --- | --- | --- | --- | --- |
|  | mean | SD | n |  | mean | SD | n |  | mean | SD | n |  | mean | SD | n |  | mean | SD | n |  | mean | SD | n |
| Pain score of shoulder pain at 24 hours | | | | | | | | | | | | | | | | | | | | | | | |
| Güngördük, 2018 | 3.90 | 0.40 | 52 |  |  |  |  |  | 2.00 | 0.40 | 54 |  |  |  |  |  |  |  |  |  |  |  |  |
| Phelps, 2008 | 2.57 | 0.47 | 46 |  |  |  |  |  |  |  |  |  |  |  |  |  | 1.08 | 0.24 | 54 |  |  |  |  |
| Ryu K, 2017 | 4.40 | 2.30 | 30 |  |  |  |  |  |  |  |  |  | 2.60 | 2.70 | 30 |  |  |  |  |  | 3.00 | 1.90 | 29 |
| Ryu KH, 2019 | 3.33 | 2.29 | 48 |  |  |  |  |  |  |  |  |  | 1.67 | 2.29 | 48 |  |  |  |  |  |  |  |  |
| Sharami, 2010 | 3.40 | 2.90 | 64 |  |  |  |  |  | 1.19 | 1.70 | 67 |  |  |  |  |  |  |  |  |  |  |  |  |
| Tsai, 2011 | 4.22 | 3.32 | 51 |  |  |  |  |  |  |  |  |  |  |  |  |  | 2.87 | 3.00 | 53 |  |  |  |  |
| Tsai, 2013 | 4.52 | 2.99 | 50 |  |  |  |  |  |  |  |  |  |  |  |  |  |  |  |  |  | 2.76 | 2.91 | 50 |
| Pain score of shoulder pain at 48 hours | | | | | | | | | | | | | | | | | | | | | | | |
| Güngördük, 2018 | 1.90 | 0.40 | 52 |  |  |  |  |  | 1.70 | 0.50 | 54 |  |  |  |  |  |  |  |  |  |  |  |  |
| Ryu K, 2017 | 3.80 | 2.00 | 30 |  |  |  |  |  |  |  |  |  | 1.40 | 1.90 | 30 |  |  |  |  |  | 2.10 | 2.00 | 29 |
| Ryu KH, 2019 | 3.17 | 2.67 | 48 |  |  |  |  |  |  |  |  |  | 1.33 | 2.29 | 48 |  |  |  |  |  |  |  |  |
| Sharami, 2010 | 1.50 | 1.66 | 64 |  |  |  |  |  | 0.46 | 0.72 | 67 |  |  |  |  |  |  |  |  |  |  |  |  |
| Tsai, 2011 | 2.20 | 2.42 | 51 |  |  |  |  |  |  |  |  |  |  |  |  |  | 1.94 | 2.73 | 53 |  |  |  |  |
| Tsai, 2013 | 3.10 | 2.88 | 50 |  |  |  |  |  |  |  |  |  |  |  |  |  |  |  |  |  | 1.76 | 2.50 | 50 |
| Study, year | **Abdominal compression** | | |  | **PRM30** | | |  | **PRM40** | | |  | **PRM40 plus IPS** | | |  | **PRM60** | | |  | **PRM60 plus IPS** | | |
|  | event |  | n |  | event |  | n |  | event |  | n |  | event |  | n |  | event |  | n |  | event |  | n |
| Incidence of shoulder pain at 24 hours | | | | | | | | | | | | | | | | | | | | | | | |
| Kumari, 2019 | 17 |  | 32 |  |  |  |  |  | 9 |  | 32 |  |  |  |  |  |  |  |  |  |  |  |  |
| Tsai, 2013 | 35 |  | 50 |  |  |  |  |  |  |  |  |  |  |  |  |  |  |  |  |  | 23 |  | 50 |
| Tsai, 2011 | 37 |  | 51 |  |  |  |  |  |  |  |  |  |  |  |  |  | 35 |  | 53 |  |  |  |  |
| van Dijk J, 2018 | 42 |  | 88 |  |  |  |  |  | 32 |  | 89 |  |  |  |  |  |  |  |  |  |  |  |  |
| Incidence of shoulder pain at 48 hours | | | | | | | | | | | | | | | | | | | | | | | |
| Kumari, 2019 | 13 |  | 32 |  |  |  |  |  |  |  |  |  | 7 |  | 32 |  |  |  |  |  |  |  |  |
| Liu, 2014 | 26 |  | 30 |  |  |  |  |  | 20 |  | 30 |  |  |  |  |  |  |  |  |  |  |  |  |
| Phelps, 2008 | 38 |  | 46 |  |  |  |  |  |  |  |  |  |  |  |  |  | 34 |  | 54 |  |  |  |  |
| Tsai, 2011 | 28 |  | 51 |  |  |  |  |  |  |  |  |  |  |  |  |  | 27 |  | 53 |  |  |  |  |
| Tsai, 2013 | 25 |  | 50 |  |  |  |  |  |  |  |  |  |  |  |  |  |  |  |  |  | 15 |  | 50 |
| van Dijk J, 2018 | 25 |  | 88 |  |  |  |  |  |  |  |  |  | 21 |  | 89 |  |  |  |  |  |  |  |  |
| Postoperative nausea/vomiting | | | | | | | | | | | | | | | | | | | | | | | |
| Güngördük, 2018 | 15 |  | 52 |  |  |  |  |  | 12 |  | 54 |  |  |  |  |  |  |  |  |  |  |  |  |
| Kumari, 2019 | 0 |  | 33 |  |  |  |  |  |  |  |  |  | 0 |  | 33 |  |  |  |  |  |  |  |  |
| Lee, 2019 | 11 |  | 42 |  | 9 |  | 42 |  |  |  |  |  |  |  |  |  |  |  |  |  |  |  |  |
| Liu, 2014 | 8 |  | 30 |  |  |  |  |  | 9 |  | 30 |  |  |  |  |  |  |  |  |  |  |  |  |
| Phelps, 2008 | 26 |  | 46 |  |  |  |  |  |  |  |  |  |  |  |  |  | 11 |  | 54 |  |  |  |  |
| Ryu KH, 2019 | 8 |  | 48 |  |  |  |  |  |  |  |  |  | 6 |  | 48 |  |  |  |  |  |  |  |  |
| Study, year | **Abdominal compression** | | |  | **PRM30** | | |  | **PRM40** | | |  | **PRM40 plus IPS** | | |  | **PRM60** | | |  | **PRM60 plus IPS** | | |
|  | event |  | n |  | event |  | n |  | event |  | n |  | event |  | n |  | event |  | n |  | event |  | n |
| Tsai, 2011 | 29 |  | 51 |  |  |  |  |  |  |  |  |  |  |  |  |  | 27 |  | 53 |  |  |  |  |
| Tsai, 2013 | 20 |  | 50 |  |  |  |  |  |  |  |  |  |  |  |  |  |  |  |  |  | 23 |  | 50 |
| van Dijk J, 2018 | 32 |  | 88 |  |  |  |  |  |  |  |  |  | 49 |  | 89 |  |  |  |  |  |  |  |  |
| Cardiopulmonary complication | | | | | | | | | | | | | | | | | | | | | | | |
| Güngördük, 2018 | 0 |  | 53 |  |  |  |  |  | 0 |  | 55 |  |  |  |  |  |  |  |  |  |  |  |  |
| Kumari, 2019 | 0 |  | 33 |  |  |  |  |  |  |  |  |  | 0 |  | 33 |  |  |  |  |  |  |  |  |
| Phelps, 2008 | 0 |  | 47 |  |  |  |  |  |  |  |  |  |  |  |  |  | 0 |  | 55 |  |  |  |  |
| Ryu K, 2017 | 3 |  | 30 |  |  |  |  |  |  |  |  |  | 5 |  | 30 |  |  |  |  |  | 1 |  | 29 |
| Ryu KH, 2019 | 2 |  | 48 |  |  |  |  |  |  |  |  |  | 4 |  | 48 |  |  |  |  |  |  |  |  |
| Sharami, 2010 | 0 |  | 65 |  |  |  |  |  | 0 |  | 68 |  |  |  |  |  |  |  |  |  |  |  |  |
| Tsai, 2011 | 0 |  | 52 |  |  |  |  |  |  |  |  |  |  |  |  |  | 0 |  | 54 |  |  |  |  |
| Tsai, 2013 | 0 |  | 51 |  |  |  |  |  |  |  |  |  |  |  |  |  |  |  |  |  | 0 |  | 51 |
| van Dijk J, 2018 | 0 |  | 89 |  |  |  |  |  |  |  |  |  | 0 |  | 90 |  |  |  |  |  |  |  |  |
| Postoperative analgesia requirement | | | | | | | | | | | | | | | | | | | | | | | |
| Güngördük, 2018 | 41 |  | 52 |  |  |  |  |  | 41 |  | 54 |  |  |  |  |  |  |  |  |  |  |  |  |
| Ryu KH, 2019 | 8 |  | 48 |  |  |  |  |  |  |  |  |  | 6 |  | 48 |  |  |  |  |  |  |  |  |
| Tsai, 2011 | 39 |  | 51 |  |  |  |  |  |  |  |  |  |  |  |  |  | 38 |  | 53 |  |  |  |  |

SD, standard deviation; n, number of participants

Abd, abdominal compression; PRM 40, pulmonary recruitment maneuver 40 cmH_2_O; PRM 60, pulmonary recruitment maneuver 60 cmH_2_O; IPS, intraperitoneal saline

**Supplement Figure S1** Risk of bias of each included study

| **Shoulder pain intensity at 24 hours**    0.0 %  87.7 %  69.2 %  50.5 %  42.6 % |
| --- |
| **Shoulder pain intensity at 48 hours**    9.8 %  42.2 %  96.5 %  74.8 %  26.7 % |
|  |
|  |

**Supplement Figure S2** SUCRA ranking of the interventions

**Supplement Table S6** Subgroup analysis according to complexity of procedures

| Treatment | Main analysis  Effect size  (95% CI) | SUCRA | | Subgroup analysis | | | | | |
| --- | --- | --- | --- | --- | --- | --- | --- | --- | --- |
|  |  |  |  | **Major**  Effect size  (95% CI) | **SUCRA** | | **Minor**  Effect size  (95% CI) | **SUCRA** | |
|  |  | **score** | **rank** |  | **score** | **rank** |  | **score** | **rank** |
| Pain score of shoulder pain at 24 hours | | | | | | | | | |
| ABD | Reference | 0.0 | 5 | Reference | 0.0 | 5 | Reference | 0.0 | 3 |
| PRM40 | -1.91  (-2.06, -1.76) | 87.7 | 1 | -1.90  (-2.06, -1.75) | 77.6 | 1 | -2.21  (-3.03, -1.39) | 97.7 | 1 |
| PRM40 plus IPS | -1.75  (-2.48, -1.02) | 69.2 | 2 | -1.90  (-3.09, -0.70) | 73.7 | 2 | Not available |  |  |
| PRM60 | -1.49  (-1.64, -1.34) | 42.6 | 4 | -1.35  (-2.57, -0.13) | 45.6 | 4 | -1.49  (-1.64, -1.34) | 52.2 | 2 |
| PRM60 plus IPS | -1.53  (-2.28, -0.78) | 50.5 | 3 | -1.57  (-2.35, -0.78) | 52.7 | 3 | Not available |  |  |
| Overall inconsistency  (p-value) | 0.86 |  |  | 0.65 |  |  | Not available |  |  |
| Number of studies | 7 |  |  | 5 |  |  | 2 |  |  |
| Pain score of shoulder pain at 48 hours | | | | | | | | | |
| ABD | Reference | 9.8 | 5 | Reference | 8.0 | 5 | Reference | 0.0 | 2 |
| PRM40 | -0.58  (-1.20, 0.05) | 42.2 | 3 | -0.20  (-0.37, -0.03) | 36.1 | 3 | -1.04  (-1.48, -0.60) | 100.0 | 1 |
| PRM40 plus IPS | -2.09  (-2.97, -1.21) | 96.5 | 1 | -2.09  (-2.77, -1.42) | 98.4 | 1 | Not available |  |  |
| PRM60 | -0.26  (-1.55, 1.03) | 26.7 | 4 | -0.26  (-1.25, 0.73) | 31.8 | 4 | Not available |  |  |
| PRM60 plus IPS | -1.46  (-2.36, -0.55) | 74.8 | 2 | -1.46  (-2.17, -0.75) | 75.9 | 2 | Not available |  |  |
| Overall inconsistency  (p-value) | 0.85 |  |  | 0.70 |  |  | Not available |  |  |
| Number of studies | 6 |  |  | 5 |  |  | 1 |  |  |
| Incidence of shoulder pain at 24 hours | | | | | | | | | |
| ABD | Reference | 8.6 | 4 | Reference | 12.3 | 3 | Reference | 1.0 | 2 |
| PRM40 | - | 76.5 | 1 | Not available |  |  | Not available |  |  |
| PRM40 plus IPS | 0.69  (0.51, 0.95) | 39.1 | 3 | Not available |  |  | 0.69  (0.51, 0.95) | 98.7 | 1 |
| PRM60 | 0.91  (0.70, 1.18) | 2.0 | 5 | 0.91  (0.70, 1.18) | 41.7 | 2 | Not available |  |  |
| PRM60 plus IPS | 0.66  (0.46, 0.93) | 58.9 | 2 | 0.66  (0.46, 0.93) | 96.0 | 1 | Not available |  |  |
| Overall inconsistency  (p-value) | Not available |  |  | Not available |  |  | Not available |  |  |
| Number of studies | 4 |  |  | 2 |  |  | 2 |  |  |

| Treatment | Main analysis  Effect size  (95% CI) | SUCRA | | Subgroup analysis | | | | | |
| --- | --- | --- | --- | --- | --- | --- | --- | --- | --- |
|  |  |  |  | **Major**  Effect size  (95% CI) | **SUCRA** | | **Minor** | **SUCRA** | |
|  |  | **score** | **rank** |  | **score** | **rank** |  | **score** | **rank** |
| Incidence of shoulder pain at 48 hours | | | | | | | | | |
| ABD | Reference | 3.7 | 5 | Reference | 18.3 | 3 | Reference | 4.2 | 4 |
| PRM40 | 0.77  (0.58, 1.03) | 54.6 | 3 | Not available |  |  | 0.77  (0.58, 1.03) | 62.2 | 3 |
| PRM40 plus IPS | 0.73  (0.48, 1.11) | 61.3 | 2 | Not available |  |  | 0.73  (0.48, 1.11) | 69.0 | 1 |
| PRM60 | 0.81  (0.66, 0.99) | 46.2 | 4 | 0.93  (0.64, 1.33) | 37.1 | 2 | 0.76  (0.60, 0.97) | 64.6 | 2 |
| PRM60 plus IPS | 0.60  (0.36, 1.00) | 84.2 | 1 | 0.60  (0.36, 0.99) | 94.5 | 1 | Not available |  |  |
| Overall inconsistency  (p-value) | Not available |  |  | Not available |  |  | Not available |  |  |
| Number of studies | 6 |  |  | 2 |  |  | 4 |  |  |

Abd, abdominal compression; PRM 40, pulmonary recruitment maneuver 40 cmH_2_O; PRM 60, pulmonary recruitment maneuver 60 cmH_2_O; IPS, intraperitoneal saline; SUCRA, surface under the cumulative ranking curve

**Supplement Table S7** Sensitivity by excluded unclear and high risk of bias study on random sequence generation and allocation concealment bias

| Treatment | Main analysis  Effect size  (95% CI) | SUCRA | | Sensitivity analysis  Effect size  (95% CI) | SUCRA | |
| --- | --- | --- | --- | --- | --- | --- |
|  |  | **score** | **rank** |  | **score** | **rank** |
| Pain score of shoulder pain at 24 hours | | | | | | |
| ABD | Reference | 0.0 | 5 | Reference | 0.3 | 5 |
| PRM40 | -1.91  (-2.06, -1.76) | 87.7 | 1 | -1.91  (-2.06, -1.76) | 83.0 | 1 |
| PRM40 plus IPS | -1.75  (-2.48, -1.02) | 69.2 | 2 | -1.75  (-2.48, -1.02) | 68.0 | 2 |
| PRM60 | -1.49  (-1.64, -1.34) | 42.6 | 4 | -1.35  (-2.57, -0.13) | 46.9 | 4 |
| PRM60 plus IPS | -1.53  (-2.28, -0.78) | 50.5 | 3 | -1.53  (-2.28, -0.78) | 51.7 | 3 |
| Overall inconsistency  (p-value) | 0.86 |  |  | 0.86 |  |  |
| Number of studies | 7 |  |  | 6 |  |  |
| Incidence of shoulder pain at 24 hours | | | | | | |
| ABD | Reference | 8.6 | 4 | Reference | 10.1 | 4 |
| PRM40 | - | 76.5 | 1 | Not available |  |  |
| PRM40 plus IPS | 0.69  (0.51, 0.95) | 39.1 | 3 | 0.75  (0.53, 1.07) | 68.0 | 2 |
| PRM60 | 0.91  (0.70, 1.18) | 2.0 | 5 | 0.91  (0.70, 1.18) | 34.4 | 3 |
| PRM60 plus IPS | 0.66  (0.46, 0.93) | 58.9 | 2 | 0.66  (0.46, 0.93) | 87.5 | 1 |
| Overall inconsistency  (p-value) | Not available |  |  | Not available |  |  |
| Number of studies | 4 |  |  | 3 |  |  |

Abd, abdominal compression; PRM 40, pulmonary recruitment maneuver 40 cmH_2_O; PRM 60, pulmonary recruitment maneuver 60 cmH_2_O; IPS, intraperitoneal saline; SUCRA, surface under the cumulative ranking curve

**Supplement Table S8** Evaluation of the global inconsistency in network meta-analysis

| Outcome | P-value for test of  global inconsistency |
| --- | --- |
| Primary outcome |  |
| Pain score of shoulder pain |  |
| - 24 hours | 0.86 |
| - 48 hours | 0.85 |
| Secondary outcome |  |
| Pulmonary complication | 0.89 |
